# Coexpression of the discoidin domain receptor 1 gene with oligodendrocyte‐related and schizophrenia risk genes in the developing and adult human brain

**DOI:** 10.1002/brb3.2309

**Published:** 2021-07-29

**Authors:** Gerard Muntané, Marc Chillida, Selena Aranda, Arcadi Navarro, Elisabet Vilella

**Affiliations:** ^1^ Department of Research Hospital Universitari Institut Pere Mata Reus Spain; ^2^ Genetics and Environment in Psychiatry Research Group Institut d'Investigació Sanitària Pere Virgili (IISPV) Reus Spain; ^3^ Department of Medicine and Surgery Universitat Rovirai Virgili (URV) Reus Spain; ^4^ Centro de investigación biomédica en red en Salud Mental (CIBERSAM) Madrid Spain; ^5^ Institute of Evolutionary Biology (IBE) Barcelona Spain; ^6^ Spanish National Research Council (CSIC) Barcelona Spain; ^7^ Department of Experimental and Health Sciences Universitat Pompeu Fabra (UPF) Barcelona Spain

**Keywords:** astrocytes, coexpression, DDR1, human brain, microglia type 2, oligodendrocytes

## Abstract

**Background:**

Discoidin domain receptor tyrosine kinase 1 (DDR1) is present in multiple types of epithelial cells and is highly expressed in the nervous system. Previous studies have revealed that *DDR1* is involved in schizophrenia (SCZ). Although the expression of *DDR1* in oligodendrocytes has been described, its role in brain myelination is not well understood. In this study, we aimed to explore the coexpression network of *DDR1* in the human brain and to compare the list of *DDR1* coexpressing genes with the list of genes containing single nucleotide polymorphisms (SNPs) that are associated with SCZ.

**Materials and Methods:**

We used a weighted gene coexpression network analysis (WGCNA) of a dataset from four brain areas (the dorsolateral prefrontal cortex, primary motor cortex, hippocampus, and striatum) and from four different intervals (I) of life (I‐1 = 10–38 weeks postconception, I‐2 ≥0 to < 6 years, I‐3 ≥ 6 to < 40 years, and I‐4 ≥ 40 years of age). We compared the list of genes that are associated with SCZ in the GWAS Catalog with the list of genes coexpressing with *DDR1* in each interval.

**Results:**

Our study revealed that *DDR1* was coexpressed with oligodendrocyte‐related genes mainly in I‐2 (adjP = 5.66e‐24) and I‐3 (adjP = 2.8e‐114), which coincided with the coexpression of *DDR1* with myelination‐related genes (adjP = 9.04e‐03 and 2.51e‐08, respectively). *DDR1* was also coexpressed with astrocyte‐related genes in I‐1 (adjP = 1.11e‐71), I‐2 (adjP = 2.12e‐20) and I‐4 (adjP = 9.93e‐52) and with type 2 microglia‐related genes in I‐1 (adjP = 2.84e‐08), I‐2 (adjP = 5.68e‐16) and I‐4 (adjP = 3.66e‐10). Moreover, we observed significant enrichment of SCZ susceptibility genes within the coexpression modules containing *DDR1* in I‐1 and I‐4 (P = 1e‐04 and 0.0037, respectively), during which the *DDR1* module showed the highest association with the astrocytes.

**Conclusions:**

Our study confirmed that *DDR1* is coexpressed with oligodendrocyte‐ and myelin‐related genes in the human brain but suggests that *DDR1* may contribute mainly to SCZ risk through its role in other glial cell types, such as astrocytes.

## Graphical abstract

- DDR1 is coexpressed with oligodendrocyte and myelin genes in the human brain
- DDR1 is coexpressed with astrocyte and microglia genes during brain development and aging
- DDR1 expression modules show enrichment with schizophrenia susceptibility genes

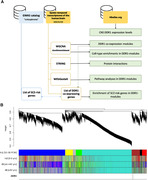

## 1 INTRODUCTION

Discoidin domain receptor tyrosine kinase 1 (DDR1) is a membrane‐anchored protein activated by fibrillar collagens (Leitinger, 2014). In adult human brain tissue, DDR1 expression is detected mainly in oligodendrocytes, but it is also found in astrocytes, activated microglia and endothelial cells (Roig et al., 2010; Vilella et al., 2019 ). DDR1 is also expressed in the peripheral nervous system, presumably in Schwann cells, and in other tissues and cell types (Leitinger, 2014; Vilella et al., 2019 ). During mouse neurodevelopment, *DDR1* is maximally expressed in oligodendrocytes throughout the myelination period (Franco‐Pons et al., 2006). It has been shown that remyelination following experimentally induced demyelination induces *DDR1* upregulation (Franco‐Pons et al., 2009). Recent single‐cell RNA‐seq studies in rodent models reported that *ddr1* expression peaks in the period in which newly formed oligodendrocytes differentiate into myelinating oligodendrocytes (Vilella et al., 2019). Data from these studies demonstrated that genes encoding the membrane receptors Ephrinb3 (Efnb3), plexinb3 (Plxnb3), ERBB3 (Erbb3) and semaphorin 4D (Sema4D); ligands (such as gelsolin [Gsn] and collagen 11 alpha 2 chain [Col11a2]); and classical myelin proteins (such as myelin‐associated oligodendrocyte basic protein [Mobp]), among others, are coexpressed with *DDR1* (Vilella et al., 2019). Nevertheless, the specific role of *DDR1* in myelination has not yet been identified.

Humans exhibit the highest level of brain myelination among mammals (including primates), which allows for a high capacity of information processing (Bartzokis, 2011). Myelin deficiencies have been observed in several psychiatric disorders, including schizophrenia (SCZ), through neuroimaging, genetic, molecular, and anatomical studies (Bartzokis, 2011; Chen et al., 2018; Koshiyama et al., 2019 ).

*DDR1* variations have been found to be associated with human schwannomas (benign tumors of myelin‐producing Schwann cells) (Agnihotri et al., 2016) and SCZ (Benkovits et al., 2016; Gas et al., 2018; Roig et al., 2007 ). Moreover, genome wide association studies (GWAS) have found associations between *DDR1* and multiple sclerosis (Mo et al., 2019), neuroticism (Kim et al., 2017), and SCZ (Pardiñas et al., 2018), although this latter study did not include the *DDR1* locus in the final analyses because it falls inside (1100 kb apart) of a linkage disequilibrium (LD) region wherein the highest SCZ‐associated locus is located. Differentially expressed levels of the DDR1c isoform (Q08345_5, 919aa) have been observed in brain tissues from patients with SCZ, compared to healthy controls, with contradictory results (Gandal et al., 2018; Roig et al., 2012 ). However, detailed studies on the coexpression of *DDR1* with other genes during human brain development are lacking.

Here, with the primary hypotheses that *DDR1* is upregulated during oligodendrocyte myelination and that *DDR1* is itself or through its interaction with other genes associated with SCZ, we aimed to identify the genes coexpressed with *DDR1* in human brain tissue in different neurodevelopmental periods and to test whether *DDR1* is coexpressed with SCZ‐associated genes.

## 2 MATERIALS AND METHODS

### 2.1 Data

Publicly available spatiotemporal transcriptome data of the human brain were used for this study (Kang et al., 2011). Raw data files were retrieved from the GEO database (GSE25219), which consisted of genome‐wide transcriptome data from 16 brain regions analyzed with the Affymetrix GeneChip Human Exon 1.0 ST Array. Samples were selected according to the following criteria: brain region, number of subjects available and tissue quality (RNA integrity number, RIN ≥8). We evaluated gene coexpression patterns in three brain regions that are involved in SCZ symptomatology, as well as one unrelated region. The first region was the dorsolateral prefrontal cortex (DFC), which is associated with high cognitive processing and was previously shown to be involved in SCZ (Friston, 1992; Lewis & Mirnics, 2006 ). The second region, the hippocampus (HIP), is involved in memory and other cognitive processes, which are impaired in SCZ (Geuze et al., 2005). The third region was the striatum (STR), which, due to its high dopaminergic activity, has been related to positive psychotic symptoms in SCZ (McCutcheon et al., 2019). Finally, the primary motor cortex (M1C), which is not associated with SCZ, was chosen as the “noncognitive” region. We grouped the 15 original periods (Kang et al., 2011) into four developmental intervals (I) considering both specific milestones in human myelination (Bartzokis, 2011; Baumann & Pham‐Dinh, 2001 ) and sample size to ensure adequate statistical power in the analyses (Iancu et al., 2012). Interval 1 (I‐1), from 10 weeks postconception (WPC) to 38 WPC (birth), coincides with the proliferation of oligodendrocyte precursor cells but low levels of myelination; Interval 2 (I‐2), from birth to < 6 years of age, is when myelination of the cognitive regions starts to increase; Interval 3 (I‐3), from ≥6 to <40 years of age, coincides with maximal myelination in cortical areas; and Interval 4 (I‐4), from ≥40 years of age on, corresponds to a decline in myelination in the brain. Ultimately, 462 brain samples (I‐1 = 188, I‐2 = 54, I‐3 = 138, and I‐4 = 82) including both hemispheres from 46 individuals were obtained. Both sexes were represented, with 22 of the subjects being female and 24 of the subjects being male (Supporting Infnormation Table S1).

All 22,011 probes were collapsed into 17,634 annotated genes. Afterwards, we filtered out the genes that did not have a normalized expression log_2_‐transformed signal intensity of ≥6 in all of the assessed areas and in all of the periods to ultimately obtain 12,643 genes.

For replication purposes, open resources from two data sets were accessed (Li et al., 2018; Zhu et al., 2018 ). Expression levels in these two studies were determined via RNA sequencing as part of the PsychENCODE (http://psychencode.org) and BrainSpan Consortia (www.brainspan.org) projects.

This study complied with the local and international standards of the ethical aspects regarding research with human data and was approved by the IISPV ethics committee.

### 2.2 Gene expression

Whole‐transcript levels of *DDR1*, classical oligodendrocyte (*OLIG2*) and myelin (*MAG, MBP*) (Baumann & Pham‐Dinh, 2001) markers and three collagen chain genes expressed in brain and which protein bind DDR1 (collagen I, *COL1A1*, and collagen IV, *COL4A1*) (Leitinger, 2014) were retrieved from the Human Brain Transcriptome database (http://hbatlas.org). These genes and other oligodendrocyte‐related (*OLIG1*, *SOX10*, *PDGFRA*, and *CSPG4*), myelin‐related (*GAC*, *CLDN11*, *CNP*, and *PLP1*), astrocyte‐related (*GFAP*) and type 2 microglia‐related (*CD53*, *CX3CL1*, and *SLC2A5*) genes were evaluated. For replication purposes, *DDR1* transcript levels were also retrieved from the Human Brain Development database (http://development.psychencode.org/).

### 2.3 Weighted gene coexpression analysis

Weighted gene coexpression network analysis (WGCNA) is a method that permits the construction of correlation networks based on gene expression patterns, allowing for the construction of modules (clusters) that correspond to genes that have highly correlated patterns of expression. Coexpression network analyses were computed independently for the four development periods using the *WGCNA* library in R software following standard protocols (Langfelder & Horvath, 2008). The weighted networks were generated by using the subset of genes with consistent levels of expression across samples. For all periods, the deep split level was set to 1 (medium sensitivity), and for each period a power (β) for which the scale‐free topology fitting index (R^2^) was ≥0.85 was selected by plotting the *R* ^2^ against soft thresholds (I‐1 = 5; I‐2 = 6; I‐3 = 6; I‐4 = 10). The maximum height at which the tree could be cut was set to 0.99, and the minimum size of the resulting modules was set at 27. The rest of the parameters were left as the default values.

The *userListEnrichment* function from the WGCNA package was used for measuring cell type enrichment between inputted and premade collections of brain‐related lists (Miller et al., 2011). Of the premade lists, only those belonging to *Human Meta* (Miller et al., 2010) were used for the enrichment function while lists from Cahoy and colleagues (Cahoy et al., 2008) were used for validation purposes. We also included one list of genes extracted from the Gene Ontology (GO) database by searching for the term “myelin” across human genes; it contained 193 genes, 175 of which were present in our curated gene set.

To enable comparisons among time intervals, each coexpression network was reassigned such that modules with significant overlap with a I‐1 module were assigned the same label. Modules without a significant overlap with modules identified in I‐1 were assigned a new label (Supporting Information Table S2).

To summarize the gene expression profiles of the highly correlated genes inside a given module, we used the first principal component, which is referred to as the module eigengene (ME). We tested each ME for correlations with the characteristics (sex, age, hemisphere, region, postmortem interval (PMI), pH, and RIN) of each sample.

To compare and integrate our gene coexpression networks with protein interaction data, we extracted protein interaction networks from the Search Tool for the Retrieval of Interacting Genes (STRING). Finally, to explore the biological functions of the *DDR1* modules, we performed GO term enrichment analysis as well as pathway ontology analyses using the REACTOME database with WebGestalt (Wang et al., 2013), and the 12,643 genes were used as background. Within each period, all P‐values were corrected using Bonferroni's multiple‐test correction.

### 2.4 Overlap with SCZ susceptibility genes

We retrieved all entries containing the word “schizophrenia” from the genome‐wide association study (GWAS) Catalog (Buniello et al., 2019) and kept the “mapped gene*”* column (accessed January 16, 2020). This yielded a total of 2,056 mapped entries, 439 of which were present in the evaluated gene dataset. We then performed 10,000 randomized bootstraps within the modules containing *DDR1* and generated random lists of genes of the same length as the module containing *DDR1*. From these bootstrapped lists, we assessed overlap with the SCZ gene set. After 10,000 iterations, we compared the distribution of bootstrapped values to the real number of genes that overlapped with SCZ in the module to obtain an empirical *p*‐value for the studied module.

## 3 RESULTS

### 3.1 *DDR1* expression

Our study focused on *DDR1* expression during brain development and in adulthood. Relative whole‐transcript *DDR1* levels in several brain regions in the I‐1 to I‐4 periods were retrieved from both the Human Brain Atlas and the Human Brain Development database, and these levels are shown in Supporting Information Figure S1. *DDR1* expression diminished over time in all the studied regions. In general, a time‐dependent decrease was observed in the expression of *COL1A1* and *COL4A1*, which code for components of collagens, the extracellular matrix ligands that activate DDR1 (Supporting Information Figure S1). However, the expression of the classical oligodendrocyte and myelin markers *OLIG2*, *CNP*, *MAG*, and *MBP* increased perinatally and was maintained at a relatively stable level until adulthood (Supporting Information Figure S1).

### 3.2 WGCNA to identify *DDR1* coexpression networks

**FIGURE 1:**
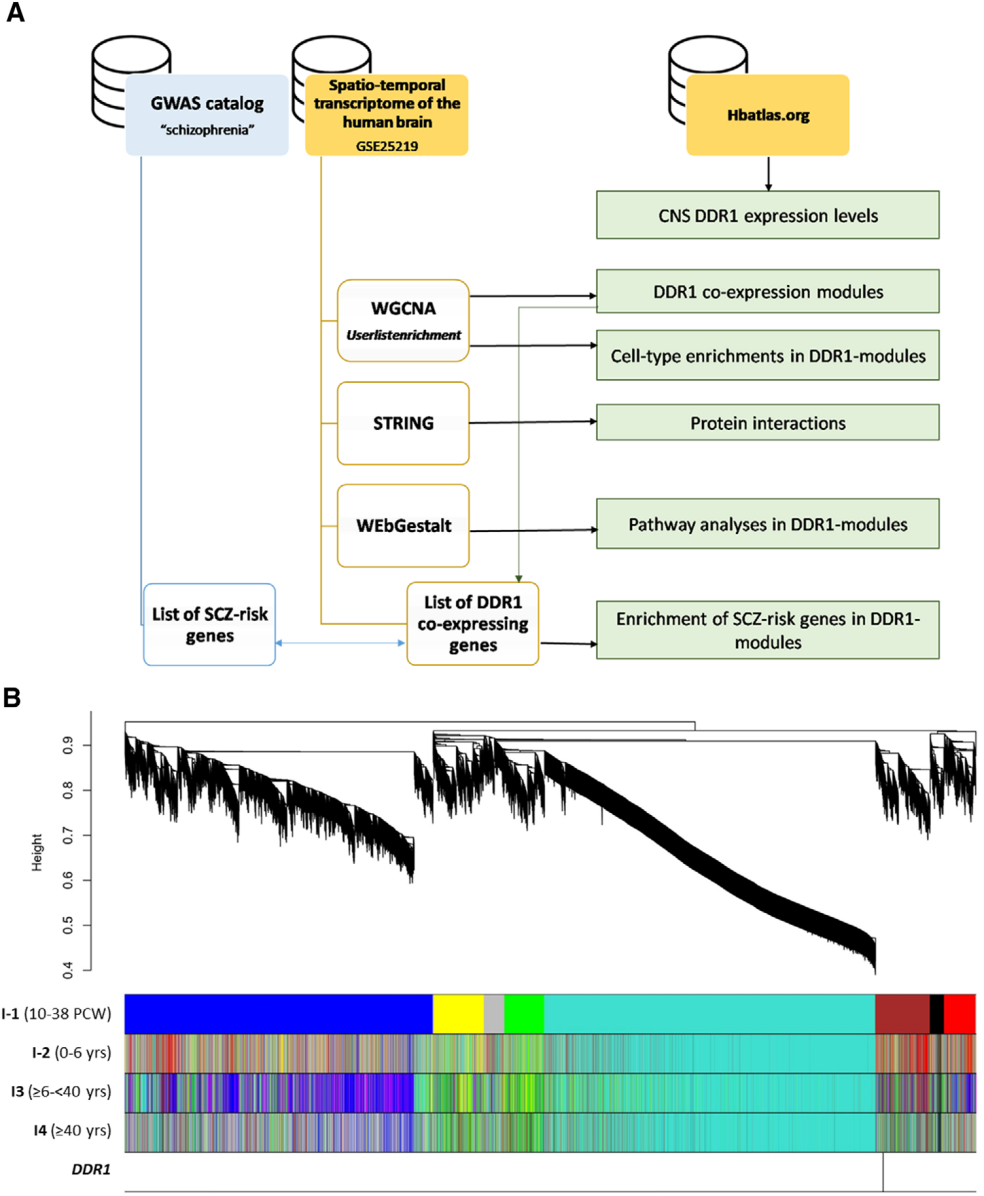
(A). Diagram of the study strategy. (B) Hierarchical clustering and visualization of gene modules in all brain periods examined in the human dataset. The network created in I‐1 served as a reference network. The module colors of other networks were redefined using the *matchLabels* function in the WGCNA R package to match the corresponding module in the human network ( Table S3). Modules of coexpressed genes were assigned colors corresponding to the branches indicated by the horizontal bars beneath the dendrogram. From top to bottom, these bars represent modules obtained using periods 1 to 4. The latter bar indicates the location of *DDR1*

WGCNA performed to identify gene coexpression networks in each development interval, and the lowest values that allowed more than 85% similarities in topological models of the four intervals were used as soft thresholds (I‐1 = 5; I‐2 = 6; I‐3 = 6; and I‐4 = 10), resulting in the detection of 8, 11, 9, and 16 modules for each of the intervals, respectively (Figure 1). Genes that were not correlated strongly enough with any module were considered background (denoted by the color gray and named hereafter as module 0, Figure 1 and Supporting Information Table S2). In I‐1, I‐2, and I‐4, *DDR1* was found in M3 (brown module) with 812, 553, and 398 genes, respectively (Supporting Information Table S3). However, *DDR1* clustered in M5 (red module) with 527 genes in I‐3 (Supporting Information Table S3). Within each period, we evaluated the correlation of the detected modules with sample traits such as sex, age, hemisphere, region, pH, PMI, and RIN. The complete list of the correlations of the modules with external traits is shown in Supporting Information Figure S2. Multiple modules were associated with one or more traits, but no module was correlated with hemisphere at any period. Although we found significant correlations with some of the external traits, we did not further analyze the data to correct for them as our study was not focused on correlation strength. All of the networks resulting from all the *DDR1* modules showed a protein‐protein interaction (PPI) enrichment value < 1e‐16 (data not shown), indicating that the proteins were strongly biologically connected.

### 3.3 *DDR1* module cell‐type enrichments

To investigate the role of *DDR1* during neurodevelopment as well as in the adult human brain, the function *userListEnrichment* was used to assess cell‐type enrichments using the human brain networks described by Miller and colleagues (Miller et al., 2010). We also evaluated the enrichment of a list of myelin‐related genes from the GO database and explored whether conventional glial cell type genes overlapped with the *DDR1* module. Table 1 shows the cell‐type enrichments in *DDR1* modules in each period, and Supporting Information Figure S3 shows the cell‐type enrichments in all modules. *DDR1* module showed coexpression with oligodendrocyte genes in all four intervals, with the highest correlation in I‐3 (P = adj1.53e‐13, adjP = 5.66e‐24, adjP = 2.18e‐114, and adjP = 1.45e‐02, respectively). Notably, in I‐3 *DDR1* was contained in M5 and the module matches exclusively with oligodendrocyte and myelin gene profiles. In I‐1, I‐2, and I‐4, *DDR1* was contained in M3 with a high correlation with astrocyte gens (adjP = 1.11e‐71, adjP = 2.12e‐20, and adjP = 9.93e‐52, respectively), microglia type 2 (adjP = 2.84e‐08, adjP = 5.68e‐16, and adjP = 3.66e‐10, respectively) and to a lesser extent to microglia type 1, which was only significant in I‐1 (adjP = 3.26e‐04). Notably, M14 in I‐4, which does not contain *DDR1*, was strongly enriched in oligodendrocyte markers (adjP = 2.62e‐60) and myelin‐related genes (adjP = 1.48e‐05) (Supporting Information Figure S3). Altogether, these results suggest that the principal role of *DDR1* in brain is in non‐mature oligodendrocytes, but some function is also associated with astrocytes and microglia.

**TABLE 1:** DDR1‐module cell‐type enrichments in each time interval

	I‐1 10 ‐ 38 PCW	I‐2 ≥0 ‐ < 6 years	I‐3 ≥6 ‐ < 40 years	I‐4 ≥ 40 years
*DDR1* module^a^	M3 (brown)	M3 (brown)	M5 (red)	M3 (brown)
Cell type module^b^				
Astrocytes	1.11e‐71	2.12e‐20	ns	9.93e‐52
Microglia (Type 1)	3.26e‐04	ns	ns	ns
Microglia (Type 2)	2.84e‐08	5.68e‐16	ns	3.66e‐10
Oligodendrocytes	1.53e‐13	5.66e‐24	2.18e‐114	1.45e‐02
Myelin^c^	ns	9.04e‐03	2.51e‐08	ns
Neuron	ns	ns	ns	ns
PVALB Interneurons	ns	ns	ns	ns
Glutamatergic Synapse	ns	ns	ns	ns
Nucleus	ns	ns	ns	ns
Mitochondria	ns	ns	ns	ns
Ribosome	ns	ns	ns	ns

^a^ *DDR1* module according to Figure 1.

^b^ Cell type modules according to Miller and colleagues (Miller et al., 2010).

^c^ Myelin genes according to GO.

ns = nonsignificant.

Conventional glial cell type gene markers and collagen genes were localized within the modules at each interval (Supporting Information Table S4). In summary, M3 at I‐1 contained classical markers for OPC, OLs‐myelin, astrocytes, and microglia. M3 in I‐2 contained gene markers for OPC, OLs‐myelin and astrocytes; M5 at I‐3 contained exclusively OL‐myelin markers; and M3 in I‐4 contained OL‐myelin and astrocyte markers. These results corroborate the cell‐type enrichments shown in Table 1. Finally, the enrichments in the *DDR1* modules were validated using mouse brain networks from Cahoy and colleagues (Cahoy et al., 2008) (Supporting Information Table S5), which confirmed that the *DDR1* module corresponded to astrocytes in I‐1 and I‐4, to astrocytes and oligodendrocytes in I‐2 and to oligodendrocytes in I‐3. Confirmatory data were retrieved from the Human Developmental Brain project, which demonstrated that DDR1 in the adult brain is mainly expressed in astrocyte and oligodendroglia cell linages (Supporting Information Figure S4).

### 3.4 Pathway enrichment in *DDR1* modules

We then performed pathway enrichment analysis of *DDR1* modules in each period (Supporting Information Tables S6–S9). The most common enriched GO term for the *DDR1* module was gliogenesis, which was significant in all periods (I‐1 to I‐4; adjP = 2.94e‐07, 5.91e‐04, 2.68e‐07, and 4.08e‐04). In I‐1, the *DDR1* module (M3) was also enriched in regulation of cell adhesion (adjP = 2.6e‐07), the ERK1 and ERK2 cascades (adjP = 1.05e‐07), and extracellular matrix organization (adjP = 1.27e‐06) (Supporting Information Table S6). In I‐2, the *DDR1* module (M3) was significantly enriched in categories such as cell substrate adhesion (adjP = 6.3e‐07) (Supporting Information Table S7). In I‐3, the *DDR1* module (M5) was highly enriched in the ensheathment of neurons (adjP = 3.13e‐06), the myelin sheath (FDR = 8.26e‐03) and the actin cytoskeleton (adjP = 8.26e‐03) (Supporting Information Table S8). Finally, in I‐4, the *DDR1* module (M3) showed enrichment in GO categories such as negative regulation of nervous system development (adjP = 4.08e‐04) (Supporting Information Table S9).

### 3.5 Overlap with SCZ risk genes

**FIGURE 2:**
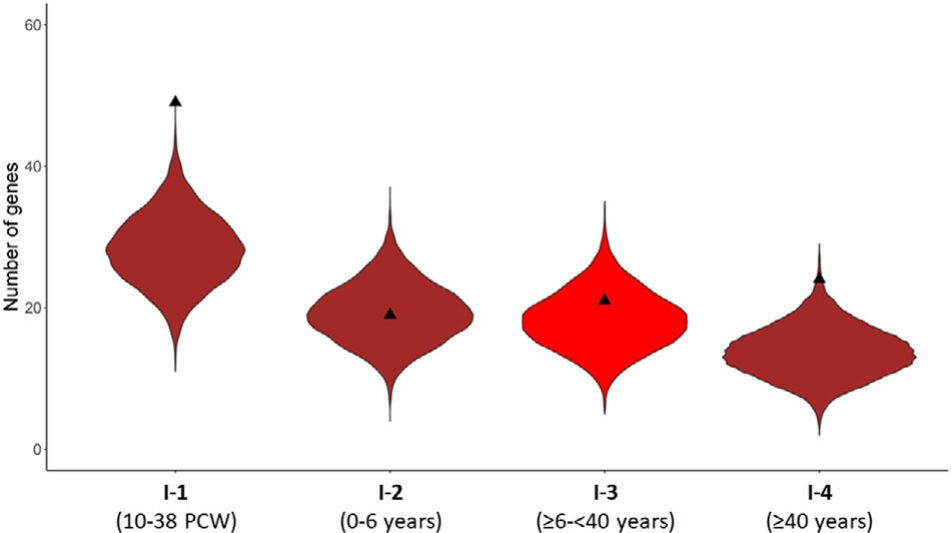
**Violin plot showing the empirical enrichment of SCZ genes in the *DDR1* module at the four periods as determined by GWAS**. Black triangles represent the observed number of overlapping genes between SCZ and *DDR1* modules in each period. The violin is colored by the DDR1 module color in each period. The violin distribution shows the number of overlapping genes after 10,000 module random bootstraps of the same gene length. In I‐1 and I‐4, the observed number of SCZ genes in the *DDR1* module was higher than expected after 10,000 bootstraps (empirical *p*‐value = 1e‐04 and 0.0037, respectively) (Supplementary Table S9)

As both *DDR1* variants and myelin impairments are involved in SCZ, we evaluated whether genes previously found by GWASs to be associated with SCZ were enriched in the *DDR1* modules in each period (Figure 2). We generated 10,000 random gene lists of the same length, one for each period (the *DDR1* module contained 812, 553, 527, and 398 genes for intervals 1, 2, 3, and 4, respectively) and evaluated the number of SCZ genes in the bootstrapped module (Supporting Information Table S10). We found that M3, which contained *DDR1* in I‐1 and I‐4, was enriched in SCZ‐associated genes (empirical *p*‐value = 1e‐04 and 0.0037, respectively). In I‐1 the overlapping list of 49 genes is represented by functions such as cell and tissue development, calcium signaling, energy metabolism, synapsis, and genes coding for proteins of the dystrophin‐associated proteins complex (DAPC). In I‐4, the overlapping list consists of 24 genes coding for proteins involved in axon growth/guidance, calcium and cell signaling, intracellular stress response and transcriptional repression. The *DDR1* modules in I‐2 and I‐3 were not enriched in SCZ susceptibility genes (empirical *p*‐values = 0.45 and 0.21, respectively).

## 4 DISCUSSION

Although *in situ* hybridization, immunohistochemical and RNA‐seq studies have mapped *DDR1* to white matter in humans and rodents (Vilella et al., 2019), a complete coexpression analysis of *DDR1* in human brain development has not been published. Here, we show that *DDR1* (whole‐transcript) is expressed at similar levels between four different brain regions (the DFC, HIP, STR, and M1C) and decreases in an age‐dependent manner (from the embryonic‐fetal stage to > 40 years of age).

The most relevant result shown here is that *DDR1*‐containing modules were significantly enriched in oligodendrocyte‐related genes in the human brain in all four‐time intervals. These results are consistent with previous findings in mice (Franco‐Pons et al., 2006; Vilella et al., 2019 ) and in the human brain (Roig et al., 2010). The highest correlation of *DDR1* expression with oligodendrocyte genes was observed in I‐3 (≥6 to < 40 years of age), followed by I‐2, I‐1, and I‐4. These results are also congruent with previous data in mice brain showing that *ddr1* expression peaks on postnatal days 15–17 (Franco‐Pons et al., 2006; Zhang et al., 2014 ), coinciding with the peak of myelination (Baumann & Pham‐Dinh, 2001). *DDR1*‐containing modules were also significantly enriched in myelin‐related genes in I‐2 and I‐3, with the highest levels being observed in I‐3, but the enrichment was lower for this module than for the oligodendrocyte module. One possible interpretation of this result, a part of a sample power explanation, is that the *DDR1* expression pattern is similar to that of oligodendrocyte markers other than myelinating oligodendrocyte markers. This interpretation is in line with the results found in mice, in which *ddr1* expression peaks between the periods in which late newly formed oligodendrocytes and myelinating oligodendrocytes are present (Marques et al., 2016; Zhang et al., 2014 ). Therefore, higher enrichment in oligodendrocyte‐related genes than myelin‐related genes (genes expressed in mature myelinating oligodendrocytes) is expected, and it suggests that in the human brain *DDR1* is relevant in differentiated oligodendrocytes that are not yet myelinating or that are myelinating but not mature. Myelin deficiencies have been observed in psychiatric disorders, such as psychosis (Mighdoll et al., 2015) and depression (Zhou et al., 2021). Recently, genome‐wide based studies have shown that myelin gene expression and regulation are altered in SCZ (Hegyi, 2017). In addition, experience‐induced myelination is known to be necessary for brain function (Fields, 2008). As an example, a recent study in mice demonstrated that remote fear memory recall depends on new myelination (Pan et al., 2020). In summary, compelling evidence supports the importance of myelin integrity for brain function and its link with psychiatric diseases.

*DDR1*‐containing modules also showed significant enrichment in microglia‐related genes, in type 2 microglia‐related genes specifically, in I‐1, I‐2, and I‐4. Likewise, *DDR1*‐containing modules were highly correlated with astrocyte‐related genes in I‐1, I‐2, and I‐4. Coexpression of *DDR1* with astrocyte‐ and microglia‐related genes could be interpreted to mean that *DDR1* is expressed in these types of cells, as has already been reported (Vilella et al., 2019). Additionally, the periods of maximal enrichment (I‐1 and I‐2) can be inferred as the developmental periods of neurogenesis, gliogenesis, synaptic formation, and synaptic pruning. All of these processes require high activity of tissue remodeling involving both astrocytes (Nutma et al., 2020) and type 2 microglia (Tang & Le, 2016). Conversely, in I‐4, aging activation of astrocytes (Boisvert et al., 2018; Clarke et al., 2018 ) and microglia (Harry, 2013) are probably related to degenerative processes associated with cognitive decline (Harry, 2013; Santello et al., 2019 ), suggesting that *DDR1* also plays a role in these cell types in this period of life. Notably, recent research has linked both astrocytes and microglia with the processes of developmental synapsis pruning and post‐developmental synaptic activity and plasticity (Mei et al., 2018; Wang et al., 2019). Moreover, in a large multicenter study of adult human brain from patients with different psychiatric disorders, the gene expression profiles of the pyramidal neurons (CA1), astrocytes and microglia cell types explained between 25% and 54% of variance in interregional profiles of group differences in cortical thickness (Patel et al., 2020).

We did not observe significant expression of neuronal genes (glutamatergic synapses, neurons, or PVALB interneurons), nuclear genes, mitochondria‐related genes or ribosome‐related genes in the *DDR1* module. In rodents, *ddr1* has been observed in proliferative and differentiating areas during neurogenesis in early neurodevelopment (Vilella et al., 2019); however, under the conditions studied here we could not detect covariations in the expression of *DDR1* and neuronal genes in the human brain, suggesting that expression of *DDR1* in fetal stages is not regulated within the main neuronal pathways.

The pathway enrichment analysis revealed that in I‐1, I‐2, and I‐4, the top pathways associated with the *DDR1* module were cell substrate adhesion and extracellular matrix interactions. However, in I‐3 (≥6‐40 years of age), the top enriched pathways were neuron ensheathment and the myelin sheath. Moreover, in I‐3, conventional myelin markers (*CNP*, *MAG*, *MBP*, *PLP1*, and *SOX10*) were found in the *DDR1* module (M5). Notably, Rho GTPase and actin binding were also significantly enriched in M5, which suggests, according to previous data (Yeh et al., 2019), that the role of DDR1 in oligodendrocytes is mediated by the Rho GTPase system and impacts actin cytoskeleton remodeling. This indicates cell movement that could be associated with oligodendrocyte process extension and the beginning of axon ensheathment.

As we previously demonstrated that SNP variants of *DDR1* are associated with SCZ (Gas et al., 2018; Roig et al., 2007 ), we also tested whether SCZ susceptibility genes are significantly more common in *DDR1*‐containing modules. We found that the overlap of the 2 lists of genes was significant in I‐1 (fetal) and I‐4 (≥40 years). Interestingly, in I‐1 and I‐4, the *DDR1* module was highly correlated with astrocytes. The neurodevelopmental hypothesis of SCZ states that disturbances in the molecular development of the brain are the first step that confers susceptibility to the disease, which manifests later in life with the outbreak of psychotic symptoms (Weinberger, 1987). Currently, this hypothesis is supported by the identification of genetic risk (Owen & O'Donovan, 2017). Based on the present data, we hypothesize that, prenatally, *DDR1* modestly contributes to SCZ risk and is mainly expressed in astrocytes and oligodendrocytes and involved in CNS architecture including synaptic pruning and myelination. In addition, *DDR1* could contribute to SCZ risk through astrocytes’ modulation of the glutamatergic neurotransmission (Mei et al., 2018). Conversely, later in adulthood *DDR1* is mainly expressed in astrocytes and microglia and could be involved in degenerative processes related to cognitive impairments also observed in chronic SCZ. This hypothesis is supported by the recent results showing that astrocyte and microglia genes as well as neuronal genes contribute to the differences in cortical thickness in schizophrenia (Patel et al., 2020). The fact that the list of genes does not significantly overlap with SCZ susceptibility genes in I‐2 and I‐3, when the *DDR1* module is highly correlated with oligodendrocyte and myelin function, suggests that myelin‐related genes do not contribute importantly to the SNP‐associated risk of SCZ development, which is in agreement with recent data pointing to neurons (Skene et al., 2018; Toker et al., 2018 ) and astrocytes (González‐Peñas et al., 2019; Toker et al., 2018 ). However, these interpretations are speculative, and further investigation is needed to address them.

Some limitations to our study exist. First, while the sample size allowed for sufficient statistical power, we could not further analyze subgroups of data based on, for instance, sex or shorter developmental time periods. Second, separate expression data for individual *DDR1* transcripts were not obtained; therefore, we could not explore which *DDR1* isoform is most associated with oligodendrocyte‐ and myelin‐related genes. Finally, WGCNA identifies clusters of genes with expression levels that are highly correlated in a given sample, but gene coexpression does not always mean that the affected genes are expressed spatially close to each other. Future functional studies assessing the exact role of *DDR1* transcripts in oligodendrocytes and other cell types in the human brain are needed.

Regarding the possible translation of these observations, Fowler and colleagues (Fowler et al., 2020) suggested that DDR1 could be a therapeutic target for neurological diseases. The author's proposal was based upon their results showing that by inhibiting DDR1, the changes in brain tissue seen in Parkinson's diseases such as inflammation, neuronal injury, autophagy and vesicular transport are reversed. Therefore, in the near future, with exhaustive description of the role of DDR1 in brain cells, the receptor can become a therapeutic target in psychiatry.

In summary, we provide convincing evidence for the involvement of *DDR1* in oligodendrocytes and for a role for this gene in myelination during human brain development. Additionally, and against our primary hypothesis, the data suggest that *DDR1* can contribute to SCZ susceptibility through coexpression with astrocyte‐related genes.

## CONFLICT OF INTEREST

All of the authors declared no conflicts of interest.

## DATA ACCESSIBILITY LINKS

For this study, we used the publicly available spatiotemporal transcriptome of the human brain dataset (Kang et al., 2011); raw data files were retrieved from the GEO database (GSE25219), https://www.ncbi.nlm.nih.gov/geo/query/acc.cgi?acc=gse25219.

## Supporting information

SUPPORTING INFORMATION

SUPPORTING INFORMATION

SUPPORTING INFORMATION

SUPPORTING INFORMATION

SUPPORTING INFORMATION
