# Supplementary figures and images for: Coexpression of the discoidin domain receptor 1 gene with oligodendrocyte‐related and schizophrenia risk genes in the developing and adult human brain
Source: Brain Behav. 2021 Jul 29;11(8):e2309. doi: 10.1002/brb3.2309 (PMC8413716; doi:10.1002/brb3.2309)

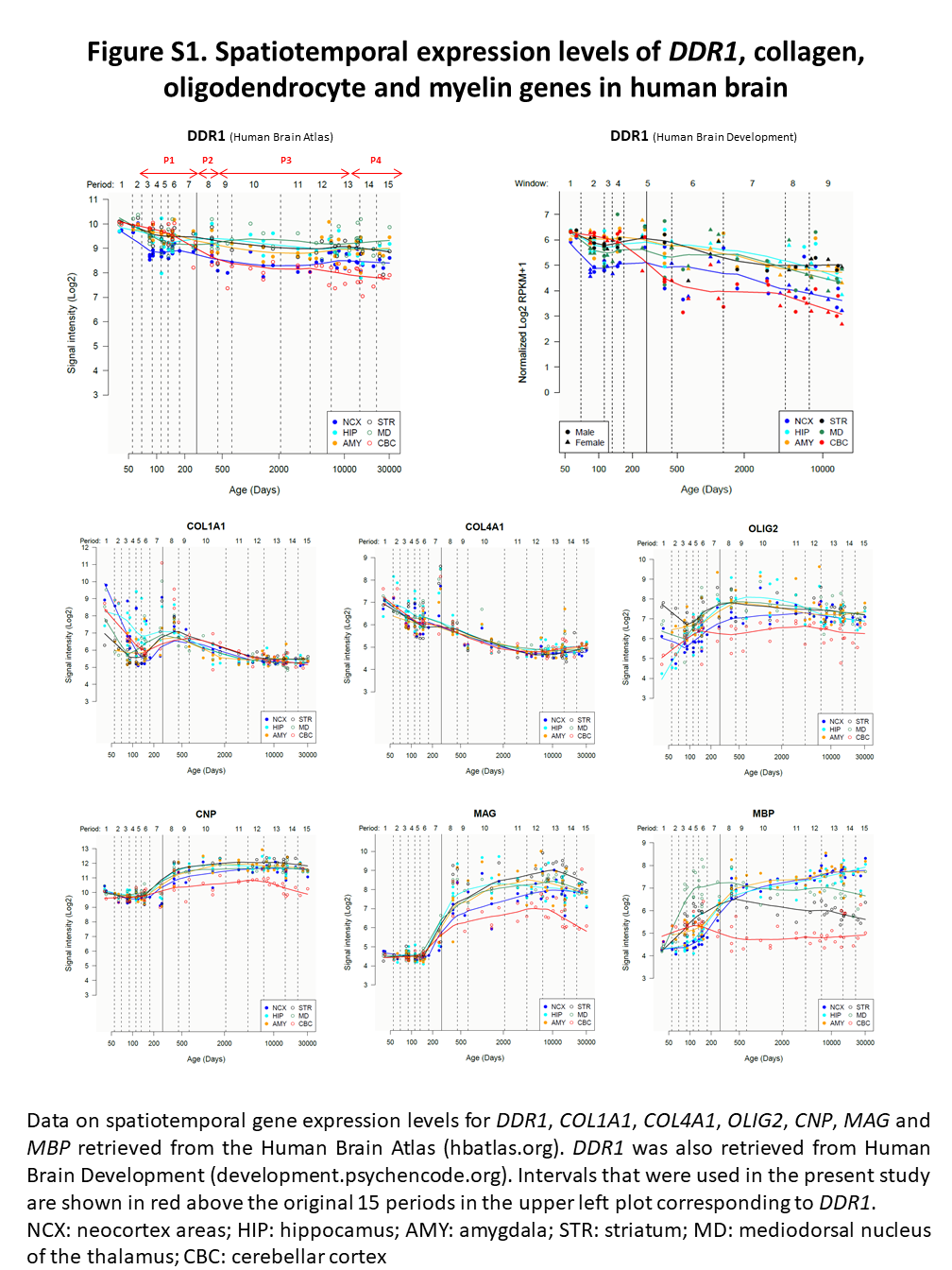

Supplement: Supplementary file 1 — SUPPORTING INFORMATION [file BRB3-11-e2309-s004.tif]

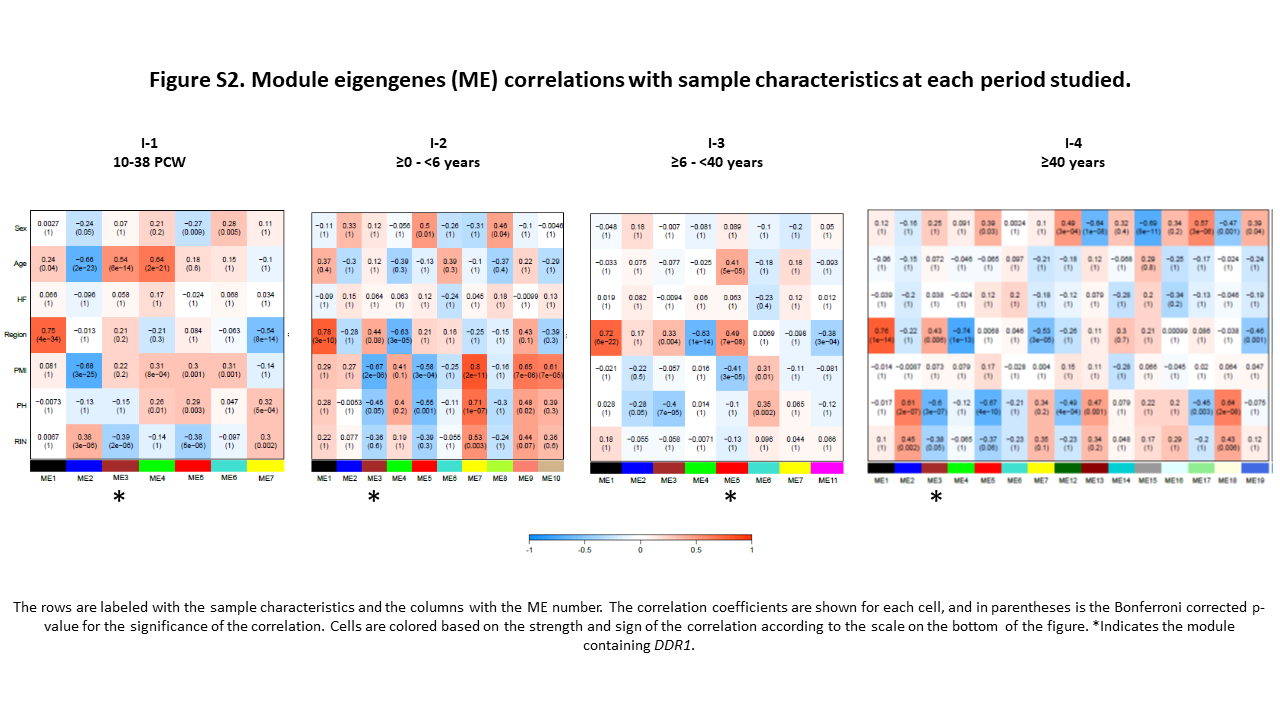

Supplement: Supplementary file 2 — SUPPORTING INFORMATION [file BRB3-11-e2309-s001.TIF]

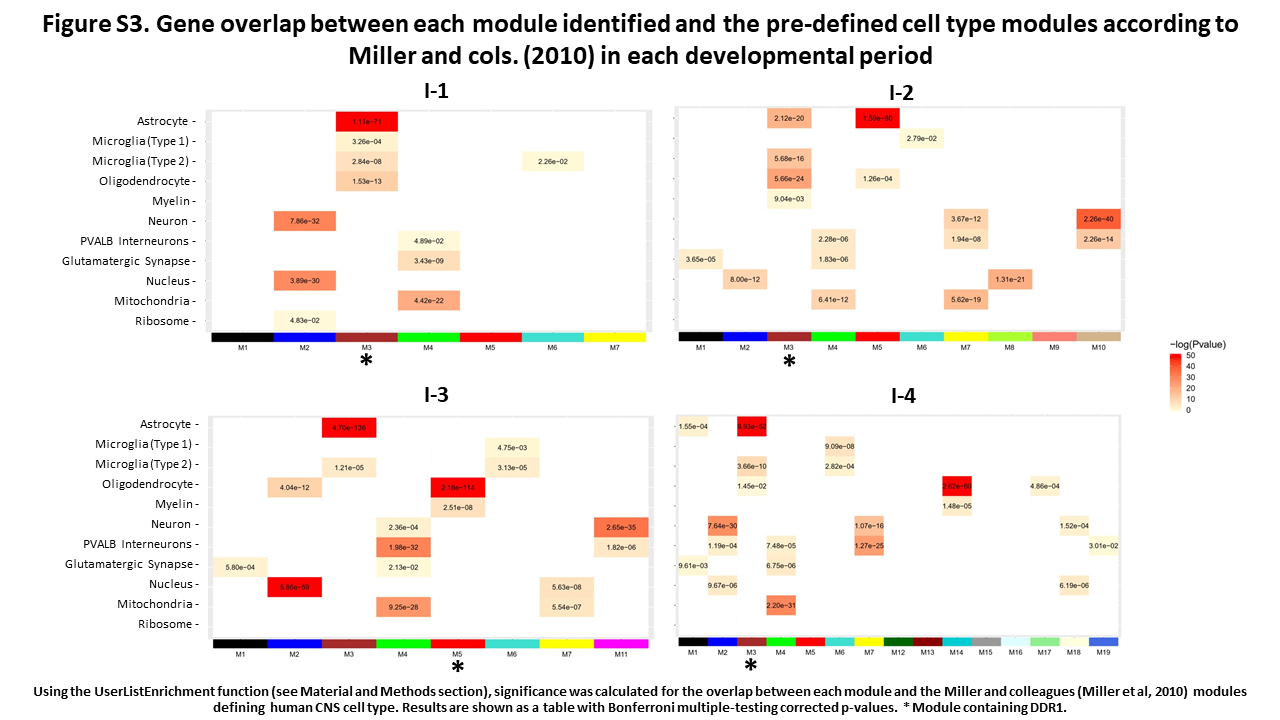

Supplement: Supplementary file 3 — SUPPORTING INFORMATION [file BRB3-11-e2309-s005.TIF]

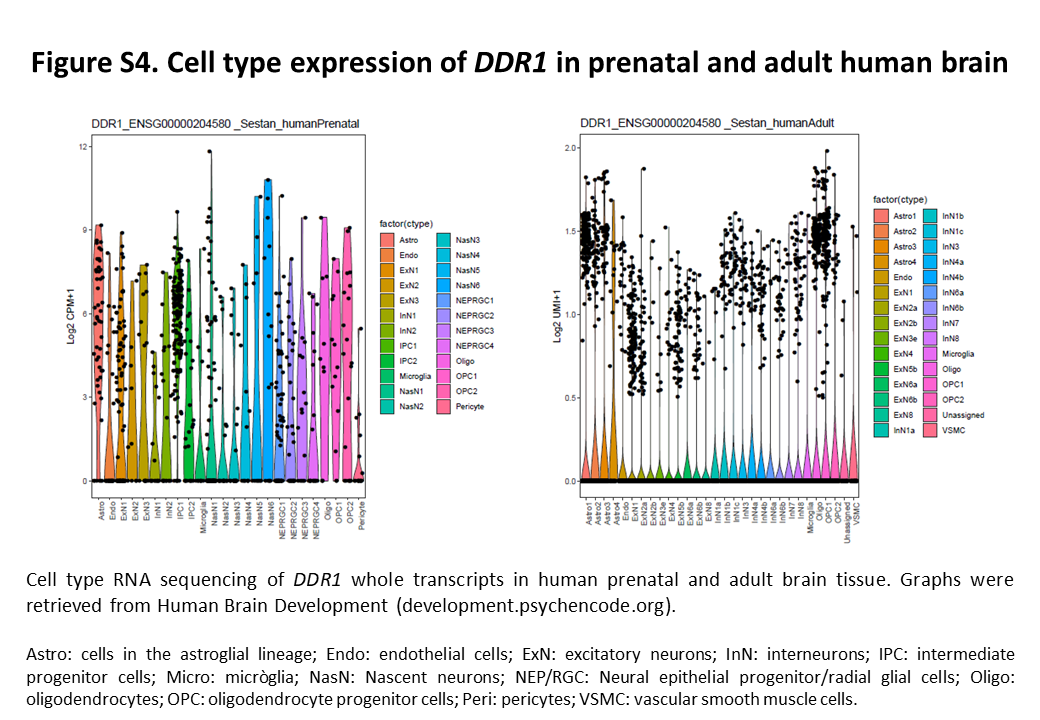

Supplement: Supplementary file 4 — SUPPORTING INFORMATION [file BRB3-11-e2309-s003.tif]
